# *Erbb4* Deletion From Inhibitory Interneurons Causes Psychosis-Relevant Neuroimaging Phenotypes

**DOI:** 10.1093/schbul/sbac192

**Published:** 2022-12-27

**Authors:** Amanda Kiemes, Maria Elisa Serrano Navacerrada, Eugene Kim, Karen Randall, Camilla Simmons, Loreto Rojo Gonzalez, Marija-Magdalena Petrinovic, David J Lythgoe, Diana Rotaru, Davide Di Censo, Lydiane Hirschler, Emmanuel L Barbier, Anthony C Vernon, James M Stone, Cathy Davies, Diana Cash, Gemma Modinos

**Affiliations:** Department of Psychosis Studies, Institute of Psychiatry, Psychology, and Neuroscience, King’s College London, London, UK; Department of Neuroimaging, School of Neuroscience, Institute of Psychiatry, Psychology, and Neuroscience, King’s College London, London, UK; Department of Neuroimaging, School of Neuroscience, Institute of Psychiatry, Psychology, and Neuroscience, King’s College London, London, UK; Department of Neuroimaging, School of Neuroscience, Institute of Psychiatry, Psychology, and Neuroscience, King’s College London, London, UK; Department of Neuroimaging, School of Neuroscience, Institute of Psychiatry, Psychology, and Neuroscience, King’s College London, London, UK; Department of Neuroimaging, School of Neuroscience, Institute of Psychiatry, Psychology, and Neuroscience, King’s College London, London, UK; MRC Centre for Neurodevelopmental Disorders, King’s College London, London, UK; Department of Forensic and Neurodevelopmental Science, Institute of Psychiatry, Psychology, and Neuroscience, King’s College London, London, UK; Department of Neuroimaging, School of Neuroscience, Institute of Psychiatry, Psychology, and Neuroscience, King’s College London, London, UK; Department of Neuroimaging, School of Neuroscience, Institute of Psychiatry, Psychology, and Neuroscience, King’s College London, London, UK; Department of Neuroimaging, School of Neuroscience, Institute of Psychiatry, Psychology, and Neuroscience, King’s College London, London, UK; Department of Psychology, University of Cambridge, Cambridge, UK; C.J. Gorter Center for High Field MRI, Department of Radiology, Leiden University Medical Center, Leiden, The Netherlands; Univ. Grenoble Alpes, Inserm, U1216, Grenoble Institut Neurosciences, Grenoble, France; Univ. Grenoble Alpes, Inserm, U1216, Grenoble Institut Neurosciences, Grenoble, France; MRC Centre for Neurodevelopmental Disorders, King’s College London, London, UK; Department of Basic and Clinical Neuroscience, School of Neuroscience, Institute of Psychiatry, Psychology, and Neuroscience, King’s College London, London, UK; Brighton and Sussex Medical School, University of Sussex, Brighton, UK; Department of Psychosis Studies, Institute of Psychiatry, Psychology, and Neuroscience, King’s College London, London, UK; Department of Neuroimaging, School of Neuroscience, Institute of Psychiatry, Psychology, and Neuroscience, King’s College London, London, UK; Department of Psychosis Studies, Institute of Psychiatry, Psychology, and Neuroscience, King’s College London, London, UK; Department of Neuroimaging, School of Neuroscience, Institute of Psychiatry, Psychology, and Neuroscience, King’s College London, London, UK; MRC Centre for Neurodevelopmental Disorders, King’s College London, London, UK

**Keywords:** mice, Erbb4, neuroimaging, inhibitory interneurons, psychosis, hippocampus

## Abstract

**Background and Hypothesis:**

Converging lines of evidence suggest that dysfunction of cortical GABAergic inhibitory interneurons is a core feature of psychosis. This dysfunction is thought to underlie neuroimaging abnormalities commonly found in patients with psychosis, particularly in the hippocampus. These include increases in resting cerebral blood flow (CBF) and glutamatergic metabolite levels, and decreases in ligand binding to GABA_A_ α5 receptors and to the synaptic density marker synaptic vesicle glycoprotein 2A (SV2A). However, direct links between inhibitory interneuron dysfunction and these neuroimaging readouts are yet to be established. Conditional deletion of a schizophrenia susceptibility gene, the tyrosine kinase receptor *Erbb4*, from cortical and hippocampal inhibitory interneurons leads to synaptic defects, and behavioral and cognitive phenotypes relevant to psychosis in mice.

**Study Design:**

Here, we investigated how this inhibitory interneuron disruption affects hippocampal *in vivo* neuroimaging readouts. Adult *Erbb4* conditional mutant mice (*Lhx6-Cre;Erbb4*^*F/F*^, *n* = 12) and their wild-type littermates (*Erbb4*^*F/F*^, *n* = 12) were scanned in a 9.4T magnetic resonance scanner to quantify CBF and glutamatergic metabolite levels (glutamine, glutamate, GABA). Subsequently, we assessed GABA_A_ receptors and SV2A density using quantitative autoradiography.

**Results:**

*Erbb4* mutant mice showed significantly elevated ventral hippccampus CBF and glutamine levels, and decreased SV2A density across hippocampus sub-regions compared to wild-type littermates. No significant GABA_A_ receptor density differences were identified.

**Conclusions:**

These findings demonstrate that specific disruption of cortical inhibitory interneurons in mice recapitulate some of the key neuroimaging findings in patients with psychosis, and link inhibitory interneuron deficits to non-invasive measures of brain function and neurochemistry that can be used across species.

## Introduction

Multiple lines of evidence suggest that GABAergic inhibitory interneuron dysfunction is a core feature of psychosis,^1^ and that this dysfunction underlies the abnormalities in brain activation commonly observed in the disorder.^2^ More specifically, *post-mortem* human brain studies in psychosis have identified reductions in the GABA-synthesizing enzyme GAD67,^3^ inhibitory interneuron number,^4^ as well as increases in GABA_A_ receptor density.^5^ Inhibitory interneuron dysfunction particularly in the hippocampus is proposed to play a critical role in psychosis pathophysiology.^6–10^ In experimental animals, hippocampal inhibitory interneuron loss has been linked to psychosis-relevant neurophysiological and cognitive deficits (i.e., reduced oscillatory activity and impaired latent inhibition).^6^ This is thought to involve a multi-synaptic pathway by which inhibitory interneuron disruption in the ventral hippocampus disinhibits glutamatergic excitatory cell activity, resulting in local hyperactivity. Glutamatergic projections from the hippocampus in turn drive increases in striatal dopamine release, proposed to underlie psychosis symptoms. A hyperactive hippocampus could also interfere with the function of hippocampal-prefrontal cortex projections, disrupting prefrontal activity, and leading to cognitive deficits.^7,8^

In humans, neuroimaging studies have identified hippocampal abnormalities consistent with a fundamental role of GABAergic inhibitory dysfunction in the pathophysiology of psychosis.^2^ Patients with psychosis exhibit hippocampal hyperactivity as indexed by increased regional cerebral blood flow (CBF)^11,12^ and cerebral blood volume (CBV),^13–17^ indirect yet highly correlated measures of neural activity due to neurovascular coupling.^18–20^ This hyperactivity has been correlated with greater severity of positive symptoms such as delusions and hallucinations.^2,7,21^ Increases in CBF are also observed in individuals at clinical high-risk (CHR) for psychosis and in healthy individuals with high schizotypy.^22–25^ Further support for GABAergic interneuron dysfunction in psychosis came from positron emission tomography (PET) research, by which antipsychotic-naïve patients with psychosis showed increases in *in vivo* GABA_A_ receptor binding with the non-selective GABA_A_ receptor (α1-3;5GABA_A_R) tracer [^11^C]flumazenil, which correlated negatively with cognition and positively with cortical EEG oscillations.^26^ More recently, studies using a more selective PET radiotracer, [^11^C]Ro15-4513, reported binding decreases in hippocampal GABA_A_ α5 receptors (α5GABA_A_R) in antipsychotic-free patients^27^ but not in patients taking antipsychotics.^27,28^ Seeking to further characterize the exact nature of hippocampal dysfunction in psychosis, reductions in the synaptic vesicle glycoprotein 2A (SV2A)—a putative marker of synaptic density^29,30^—have been reported in the hippocampus of patients by *in vivo* [^11^C]UCB-J PET imaging.^31,32^ Finally, studies using proton magnetic resonance spectroscopy (^1^H-MRS) identified increases in the levels of combined glutamine and glutamate (Glx),^33,34^ but not GABA,^35,36^ in the hippocampus of patients with psychosis compared to healthy controls. Despite these recent human neuroimaging advances enabling non-invasive investigation of GABAergic dysfunction in psychosis, *in vivo* neuroimaging assessments cannot directly inform whether these signal changes are associated with inhibitory interneuron function.

One way to address the issue of cellular specificity is by targeted (e.g., genetic) modification of specific cell types in experimental animals. This allows the effects of such genetic modifications to be assessed using the same neuroimaging modalities used in human studies,^37,38^ providing more direct evidence to link cellular defects to macroscopic *in vivo* neuroimaging changes. For example, previous work in the cyclin D2 knockout mouse model identified increased CBV as a result of hippocampal PV+ interneuron reduction.^39^ Furthermore, deletion of tyrosine kinase receptor *Erbb4* (a susceptibility gene linked to psychosis^40,41^) from inhibitory interneurons^42,43^ in the cortex and hippocampus in mice was demonstrated to lead to a constellation of psychosis-relevant biomarkers.^44–47^ These include pre- and post-synaptic deficits in PV+ interneurons (e.g., decreased interneuron signaling in the hippocampus, and dysregulated activity of hippocampal pyramidal cells),^44^ elevated striatal dopamine^47^ and psychosis-relevant behaviors (e.g., hyperlocomotion, impaired pre-pulse inhibition, impaired cognitive, and social behavior).^44^*Erbb4* mutant mice thus represent a suitable model with which to analyze the contribution of inhibitory interneuron dysfunction to neuroimaging-based markers of hippocampal dysfunction associated with psychosis in humans using non-invasive, clinically translational methods.

Here, we sought to determine how inhibitory interneuron dysfunction in *Erbb4* mutants affects *in vivo* neuroimaging readouts commonly used in psychosis research: arterial spin labeling (ASL) to measure CBF, and ^1^H-MRS to measure glutamate, glutamine, and GABA levels in the hippocampus. Next, we sought to characterize hippocampal receptor and synaptic densities in this model, using *ex vivo* quantitative autoradiography with radioligands previously used in human *in vivo* PET studies: [^3^H]Ro15-4513 to measure α5GABA_A_R, [^3^H]flumazenil for α1-3;5GABA_A_R, and [^3^H]UCB-J for SV2A. Based on the synaptic deficits previously reported in these animals,^44^ and the evidence that inhibitory interneuron deficits may underlie hippocampal hyperactivity in psychosis,^2^ we hypothesized that *Erbb4* mouse mutants would show increases in CBF, glutamatergic metabolites and α1-3;5GABA_A_R density, as well as decreases in α5GABA_A_R and SV2A density, in the hippocampus compared to wild-type littermate controls.

## Methods

### Animals

All animal procedures were performed in accordance with UK Home Office Animals (Scientific Procedures) Act 1986 and approved by the local King’s College London Animal Welfare Ethical Review Body. Animals were maintained under standard laboratory conditions on a 12:12 h light/dark cycle with water and food ad libitum. Mice carrying loxP-flanked *Erbb4* alleles^45^ were crossed with *Lhx6-Cre* mice^48^ to generate *Lhx6-Cre;Erbb4*^*F/F*^ conditional mutants. Of the interneurons expressing the transcription factor *Lhx6*,^48^*Erbb4* is primarily expressed on PV+ interneurons, and to a negligible amount on somatostatin- and calretinin-expressing interneurons.^42–44,49^ Thus, *Lhx6-Cre;Erbb4*^*F/F*^ mice exhibit *Erbb4* deletion primarily in PV+ interneurons. Wild-type *Erbb4*^*F/F*^ littermates were used as controls.

### Experimental Design

Twelve *Lhx6-Cre;ErbB4*^*F/F*^ (9 female; 3 male) and 12 *Erbb4*^*F/F*^ control (5 female; 7 male) adult (PD98 ± 11 days) mice underwent approximately 2 h of *in vivo* MR imaging. MR images were acquired using a 9.4T Bruker BioSpec 94/20 scanner with an 86-mm volume transmission coil and receive-only 2 × 2 surface array coil. All MR data were acquired from anesthetized animals (see “Anesthesia” section) in a single scanning session. Brains were collected immediately after scanning for quantitative autoradiography.

### Anesthesia

Mice were initially anesthetized with 5% isoflurane in a mixture of 70% air and 30% oxygen. After positioning on the scanner bed, a subcutaneous bolus of medetomidine (0.05 mg/kg) was administered and the isoflurane was reduced to 1.5%. Eight minutes after the bolus, a subcutaneous infusion of medetomidine (0.1 mg/kg/h) was started and maintained until the end of the ASL scan.^50,51^ Then, the medetomidine infusion was stopped and the isoflurane level was increased to 2% for the remaining scans.

### Arterial Spin Labeling

Pseudo-continuous ASL (pCASL) was used to quantify CBF. The pCASL protocol includes a perfusion scan and 2 pre-scans to determine the optimal label and control phase increments and an inversion efficiency (IE) scan for each mouse.^52^ The labeling slice was positioned 5 mm upstream of the carotid bifurcation. The labeling duration (τ) and post-label delay were 3000/300 ms, 1500/300 ms, and 200/0 ms for the perfusion scan, pre-scans, and IE scan, respectively. The pre-scans and perfusion scan used a 2D spin-echo echo-planar imaging readout: echo time (TE)/repetition time (TR) = 14.1/4000 ms, readout bandwidth = 300 kHz, matrix = 92 × 60, field-of-view (FOV)=18.4 × 12 mm. Ten 1-mm-thick slices were acquired for the perfusion scan, and a single 4 mm-thick slice for the pre-scans. For the IE scan, a single 1 mm-thick slice 3 mm downstream of the labeling slice was acquired using a flow-compensated gradient echo sequence: TE/TR = 5.2/220 ms, flip angle (FA) = 25°, matrix = 200 × 180, FOV = 20 × 18 mm, 4 averages. The perfusion scan comprised 40 label/control image pairs. Four additional control images were acquired with reversed phase-encoding blips for distortion correction, which was performed using FSL topup (v5.0.10^53^).

Second, T1 maps were acquired for CBF quantification using an MP2RAGE sequence: TE/TR = 2.5/7 ms, MP2RAGE_TR_ = 7000 ms, inversion times TI1/TI2 = 800/2500 ms, FA = 7/7°, matrix = 108 × 108 × 64, FOV = 16.2 × 16.2 × 9.6 mm. The qi_mp2rage command from the QUantitative Imaging Toolbox (QUIT v2.0.2^54^) was used to compute *T*1 maps from the complex MP2RAGE images.

Custom MATLAB scripts were written to calculate the mean IE in manually drawn regions of interest (ROIs) around both carotid arteries and quantitative CBF maps using the following equations:


$$\begin{array}{l} IE=\frac{\left|M_{control}\;-\;M_{label}\right|}{2M_{control}} \end{array}$$

$$\begin{array}{l} CBF=\frac{6000\;\cdot \;\lambda \;\cdot \;\left(SI_{control}\;-\;SI_{label}\right)\;\cdot \;e^{PLD/T1_{blood}}}{2\;\cdot \;IE\;\cdot \;T1_{blood}\;\cdot \;SI_{control}\;/\;\left(1\;-\;e^{-TR/T1}\right)\;\cdot \;\left(1\;-\;e^{\tau /T1_{blood}}\right)} \end{array}$$

*M*_control_ and *M*_label_ are the complex signals from the control and label images from the IE scan, SI_control_, and SI_label_ are the time-averaged signal intensities of the control and label images from the perfusion scan, assuming the blood-brain partition coefficient *λ* = 0.9 ml/g, and *T*1_blood_ = 2.4 s.

**Fig. 1.**
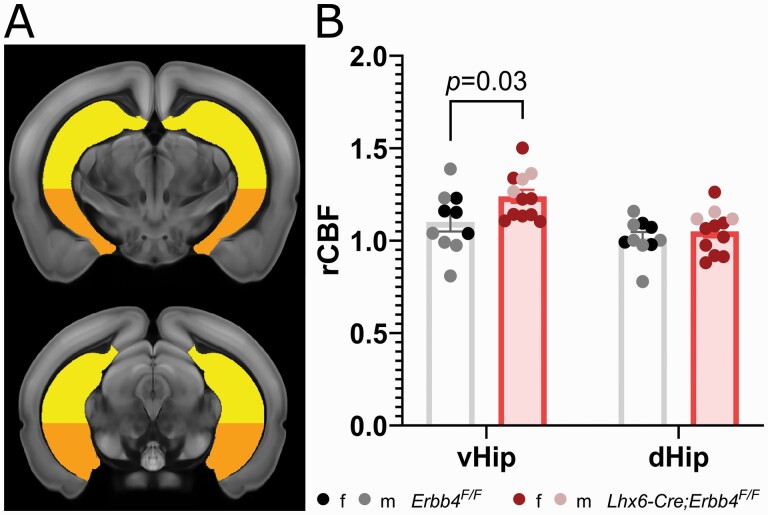
Regional CBF in *Lhx6-Cre;Erbb4*^*F/F*^ mice is increased in the ventral hippocampus. (A) Hippocampal regions of interest overlaid on a standard mouse brain template (approximate distance from Bregma,^56^ top −2.8, bottom −3.2); yellow (top) = dorsal hippocampus, orange (bottom) = ventral hippocampus. (B) Greater CBF in the ventral (*P*_corr_ = 0.03, *d *= 0.80), but not dorsal hippocampus (*P*_corr_ > 0.9, *d *= 0.21) of *Lhx6-Cre;Erbb4*^*F/F*^ mutants (*n* = 12, 9 female, 3 male) compared to control mice (*n* = 10, 4 female, 6 male). vHip: ventral hippocampus; dHip: dorsal hippocampus.

The T1 images were used to register all subjects to the Allen mouse brain Common Coordinate Framework v3 (CCFv3) using antsRegistration to perform sequential rigid-body, affine, and SyN diffeomorphic registrations (ANTs v2.1.0^55^). As there were no differences between the genotypes in whole brain CBF (mean ± SD ml/100 g/min, 62.4 ± 19.9 *Erbb4*^*F/F*^ vs 59.7 ± 11.6 *Lhx6-Cre;Erbb4*^*F/F*^, *P *= .69, 2-tailed *t*-test) CBF maps were normalized by the mean whole brain CBF, and then mean regional CBF/whole brain CBF ratio values were calculated for 21 ROIs derived from the CCFv3 atlas labels. We focused our analyses on the dorsal and ventral hippocampus (figure 1A). For completeness, exploratory independent *t*-tests of other atlas-derived ROIs are presented in the supplementary table S2.

### Magnetic Resonance Spectroscopy

**Fig. 2.**
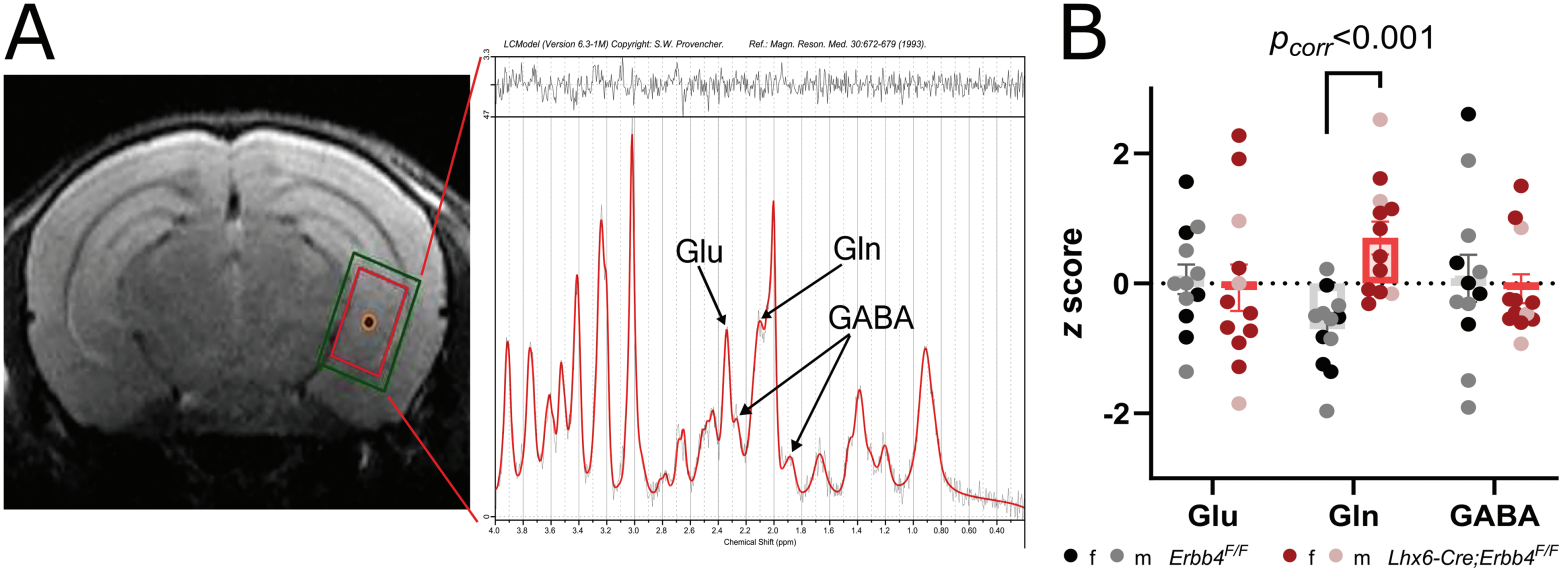
(A) Representative ^1^H-MRS PRESS voxel (red; inner rectangle) and corresponding shim (green; outer rectangle) placement in ventral hippocampus (left) and ^1^H-MRS spectrum (right). (B) *Z*-scores of ^1^H-MRS metabolites in the ventral hippocampus. Glutamine was significantly higher in *Lhx6-Cre;Erbb4*^*F/F*^ mutant mice (*n* = 12, 9 female, 3 male) compared to control mice (*n* = 12, 5 female, 7 male; *P*_*corr*_ < .001, *d *= 1.96). GABA: gamma-aminobutyric acid; Glu: glutamate; Gln: glutamine.

Finally, ^1^H-MRS was used to quantify hippocampal metabolite profiles^57^ in conditional *Erbb4* mouse mutants and controls. After manually placing the voxel on the hippocampus (figure 2A) with the aid of *T*1 structural images, individual spectra were acquired using a Point REsolved Spectroscopy (PRESS) pulse sequence^58^ with the following parameters: TE = 8.23 ms, TR = 2500 ms, 512 averages, acquisition bandwidth = 4401 Hz, 2048 acquisition points, voxel size = 1.5 × 1.5 × 3 mm. Outer volume suppression and water suppression with variable pulse power and optimized relaxation delays (VAPOR) were used in order to mitigate the contribution of signal from outside the prescribed voxel and suppress unwanted signal from water.

To analyze MR spectra, first, FID Appliance (FID-A^59^) was used to preprocess ^1^H-MRS data, simulate the metabolites, and create a basis set (model spectra). Then, we used Linear Combination (LC) Model version 6.3^60,61^ to calculate the water-referenced concentration (in mM) of the different metabolites by applying linear combinations of the model spectra to determine the best fit of the individual ^1^H-MRS data.^62^ Finally, the method of Cramér Rao (Cramér Rao Lower Bound, CRLB) was applied to ensure the reliability of the metabolite quantification, by which metabolite concentrations with S.D. ≥20% are classified as not accurately detectable and are discarded.^63,64^ Using these criteria no data had to be discarded (see quality control parameters in supplementary table S3) for our metabolites of interest: gamma-aminobutyric acid (GABA), glutamine (Gln), and glutamate (Glu) (figure 2).

### Quantitative Autoradiography

Following MR scanning, the mice were transcardially perfused with ice-cold heparinized (50 iu/ml) saline (0.9% NaCl in dH_2_O), the brains dissected, and flash-frozen in cold (ca. −40°C) isopentane on dry ice, then stored at −80°C. Frozen brains were coronally cryosectioned at 20 µm and mounted onto glass slides, then dried on a hotplate. Quantitative autoradiography was performed as previously described^65,66^ using radioligands [^3^H]Ro15-4513, [^3^H]flumazenil, and [^3^H]UCB-J. All slides were immersed in Tris buffer (50 mM) for 20 min prior to incubation with radioligands for specific or nonspecific binding, and incubation was followed by 2 washes in Tris buffer for 2 min each, and a rinse in dH_2_O, before overnight air-drying.

To quantify density of α5GABA_A_R,^67–69^ sections were incubated for 60 min at room temperature in 2 nM [^3^H]Ro15-4513 (Perkin Elmer, NET925250UC), or in 2nM [^3^H]Ro15-4513 with 10 µM bretazenil (Sigma, B6434) for nonspecific binding. To quantify α1-3;5GABA_A_R^70^ sections were incubated for 60 min at 4°C in 1 nM [^3^H]flumazenil (Perkin Elmer, NET757001MC), or in 1 nM [^3^H]flumazenil with 10 µM flunitrazepam (Sigma Aldrich, F-907 1ML) for nonspecific binding. To quantify SV2A density,^71^ sections were incubated for 60 min at room temperature in 3 nM [^3^H]UCB-J (Novandi Chemistry AB, NT1099), or in 3 nM [^3^H]UCB-J with 1mM levetiracetam (Sigma Aldrich, L8668) for nonspecific binding.

Dried slides and [^3^H] standards (American Radiolabelled Chemicals, Inc., USA, ART-123A) were placed into light-proof cassettes, and a [^3^H]-sensitive film (Amersham Hyperfilm, 28906845) was placed on top. The films were exposed 2 weeks for [^3^H]UCB-J, 4 weeks for [^3^H]flumazenil and 8 weeks for [^3^H]Ro15-4513. All films were developed with an Optimax 2010 film developer (Protec GmbH & Co, Germany) and autoradiographs captured using an AF-S Micro NIKKOR 60 mm lens on top of a light box (Northern Lights, USA). Lighting conditions were kept the same during imaging capture of each film. Optical density was measured in standards and ROIs of autoradiographs using ImageJ (1.52e). Nonspecific binding was absent for [^3^H]Ro15-4513 and [^3^H]flumazenil (supplementary figure S1). [^3^H]UCB-J nonspecific binding was minimal and checked for group differences (supplementary figure S1). Specific receptor binding (µCi/mg) was calculated with robust regression interpolation in GraphPad Prism (v9.2.0 for Windows) using standard curves created from optical density measurements of [^3^H]-standards slide for each film.

**Fig. 3.**
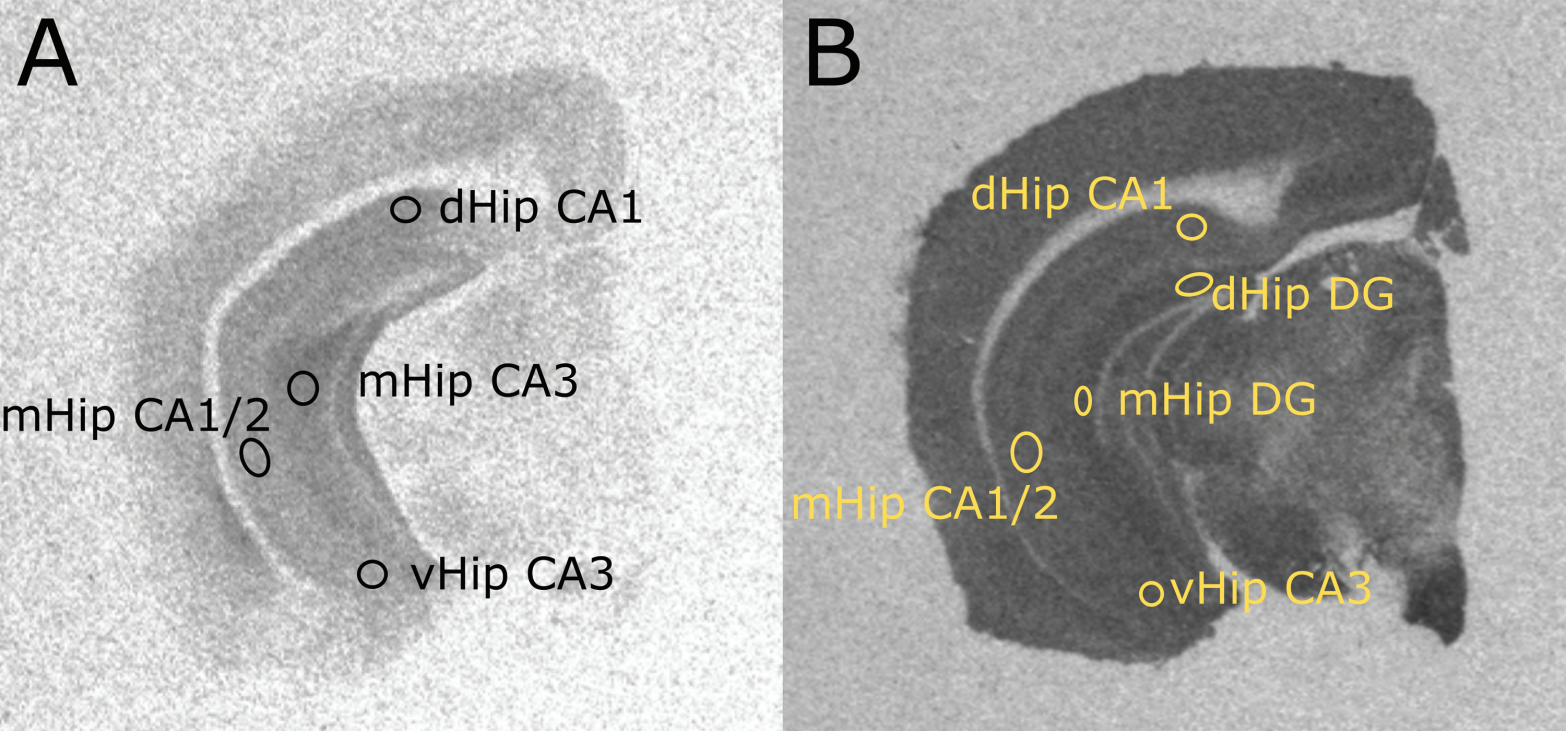
Representative hippocampal regions of interests for quantitative autoradiography. (A) Regions of interests sampled for [^3^H]Ro15-4513 and [^3^H]flumazenil. (B) Regions of interests sampled for [^3^H]UCB-J. dHip CA1: dorsal hippocampus CA1; mHip CA3: middle hippocampus CA3; mHip CA1/2: middle hippocampus CA1/CA2; vHip CA3: ventral hippocampus CA3; dHip DG: dorsal hippocampus dentate gyrus; mHip DG: middle hippocampus dentate gyrus.

All regions of interest (ROI) were sampled and averaged from three consecutive brain sections per mouse, for all radioligands. For [^3^H]Ro15-4513 and [^3^H]flumazenil, 4 ROIs were sampled (figure 3A): CA1 of the dorsal hippocampus, CA3 of the middle hippocampus, CA1/2 of the middle hippocampus, and the CA3 of the ventral hippocampus. Owing to better signal/contrast to noise ratio of [^3^H]UCB-J autoradiographs (figure 3B), we also analyzed the binding in the dentate gyrus. These hippocampal ROIs were selected based on previous evidence implicating their involvement in psychosis^13,16,21,44,72,73^ and defined using the Paxinos and Franklin’s mouse brain atlas.^56^ For completeness, further non-hippocampal ROIs (amygdala, retrosplenial cortex, visual cortex, prelimbic cortex, motor cortex, orbital cortex; supplementary figure S2) were sampled, and their exploratory statistical analyses for all 3 radioligands are presented in the supplementary tables S4–6).

### Statistical Analysis

Statistical analysis was conducted using GraphPad Prism software (v9.2.0 for Windows). To investigate the group differences in CBF and autoradiography data, we used a mixed-effects model, with the genotype (*Lhx6-Cre;Erbb4*^*F/F*^, or *Erbb4*^*F/F*^) as between-group factor and ROI as within-group factor. Any significant genotype × ROI interaction was followed up by Bonferroni-adjusted post-hoc tests (adjusted *P*-values then reported as *P*_corr_). For metabolite data, groups were compared by independent *t*-tests and *P*-values were Bonferroni-corrected in R (v1.3.1093) and reported as *P*_corr_. To better graphically depict the comparison between different ^1^H-MRS metabolites, we calculated *z* scores of individual concentrations in relation to the pooled group mean metabolite concentration. In addition, supplementary mixed ANCOVA analyses were run in IBM SPSS for Windows Version 28.0.1.1^74^ to investigate potential effects of sex or CRLB values on significant findings. Cohen’s *d* and η^2^ effect sizes were calculated from test statistics using the effectsize library (v0.5^75^) in R (v1.3.1093). Significance threshold was set to *P *< .05.

Due to technical failures (i.e., scanning faults, inadequate tissue preparation), and Covid-19 restrictions limiting laboratory access, the following mouse data were missing: 2 *Erbb4*^*F/F*^ mice for CBF, 3 *Erbb4*^*F/F*^ and 1 *Lhx6-Cre;Erbb4*^*F/F*^ mice for [^3^H]UCB-J, 1 *Erbb4*^*F/F*^ and 2 *Lhx6-Cre;Erbb4*^*F/F*^ mice for [^3^H]-Ro15-4513, and 2 *Erbb4*^*F/F*^ and 3 *Lhx6-Cre;Erbb4*^*F/F*^ mice for [^3^H]flumazenil (see supplementary table S1 for detailed *n*’s).

## Results

### Ventral Hippocampal CBF is Increased in Lhx6-Cre;Erbb4F/F Mice

A mixed-effects model investigating the effect of genotype (*Erbb4*^*F/F*^ vs *Lhx6-Cre;Erbb4*^*F/*F^) and ROI (ventral vs dorsal hippocampus) on CBF revealed no significant main effect of genotype (*F*_(1,20)_ = 2.78, *P *= .11, η^2^ = 0.12). We observed a significant main effect of ROI (*F*_(1,20)_ = 87.96, *P *< .001, η^2^ = 0.81) and a significant genotype × ROI interaction (*F*_(1,20)_ = 11.91, *P *= .003, η^2^ = 0.37). Follow-up analysis (figure 1B) revealed that this was due to a significant CBF increase in *Lhx6-Cre;Erbb4*^*F/F*^ mice compared to controls in the ventral (*t*_(40)_ = 2.54, *P*_corr_ = .03, *d *= 0.80) but not dorsal hippocampus (*t*_(40)_ = 0.67, *P*_corr_ > 0.9, *d *= 0.21). These effects remained unchanged after adding sex as a covariate of no interest in the analysis (genotype × ROI interaction effect: *F*_(1,19)_ = 8.75, *P *= .008, η^2^ = 0.32; ventral CBF increase: *F*_(1,19)_ = 4.52, *P*_corr_ = .047, η^2^ = 0.19).

### Glutamine Levels are Increased in Ventral Hippocampus of Lhx6-Cre;Erbb4^F/F^ Mice

*Lhx6-Cre;Erbb4* ^*F/F*^ mice showed significantly higher glutamine concentration compared to control animals (*t*_(22)_ = 4.60, *P*_corr_ < .001, *d *= 1.96, figure 2B and table 1). As glutamine CRLB varied significantly between the 2 genotype groups (supplementary table S3), we ran a supplementary ANCOVA controlling for glutamine CRLB and sex, by which the group difference remained significant (*F*_(1,20)_ = 20.54, *P *< .001, η^2^ = 0.51). There were no significant group differences in either glutamate or GABA concentrations (table 1).

**Table 1.** ^1^H-MRS Absolute Metabolite Concentrations in Millimolar

	*Erbb4* ^*F/F*^ (*n* = 12)	*Lhx6-Cre;Erbb4* ^*F/F*^ (*n* = 12)	*Erbb4* ^*F/F*^ vs *Lhx6-Cre;Erbb4*^*F/F*^	
	Mean (SD)	Mean (SD)	*t*	*P*_corr_
Glu	6.77 (0.51)	6.68 (0.80)	0.31	>.9
Gln	2.29 (0.23)	2.84 (0.34)	4.60	<.001
Glx	9.04 (0.64)	9.61 (0.84)	1.86	.15
GABA	2.08 (0.49)	2.02 (0.30)	0.38	>.9

*Note*: Glu: glutamate; Gln: glutamine; Glx: glutamate + glutamine; *P*_corr_: Bonferroni-adjusted *P* values

### Lhx6-Cre;Erbb4^F/F^ Mice Display Decreased [^3^H]UCB-J Binding in the Hippocampus

**Fig. 4.**
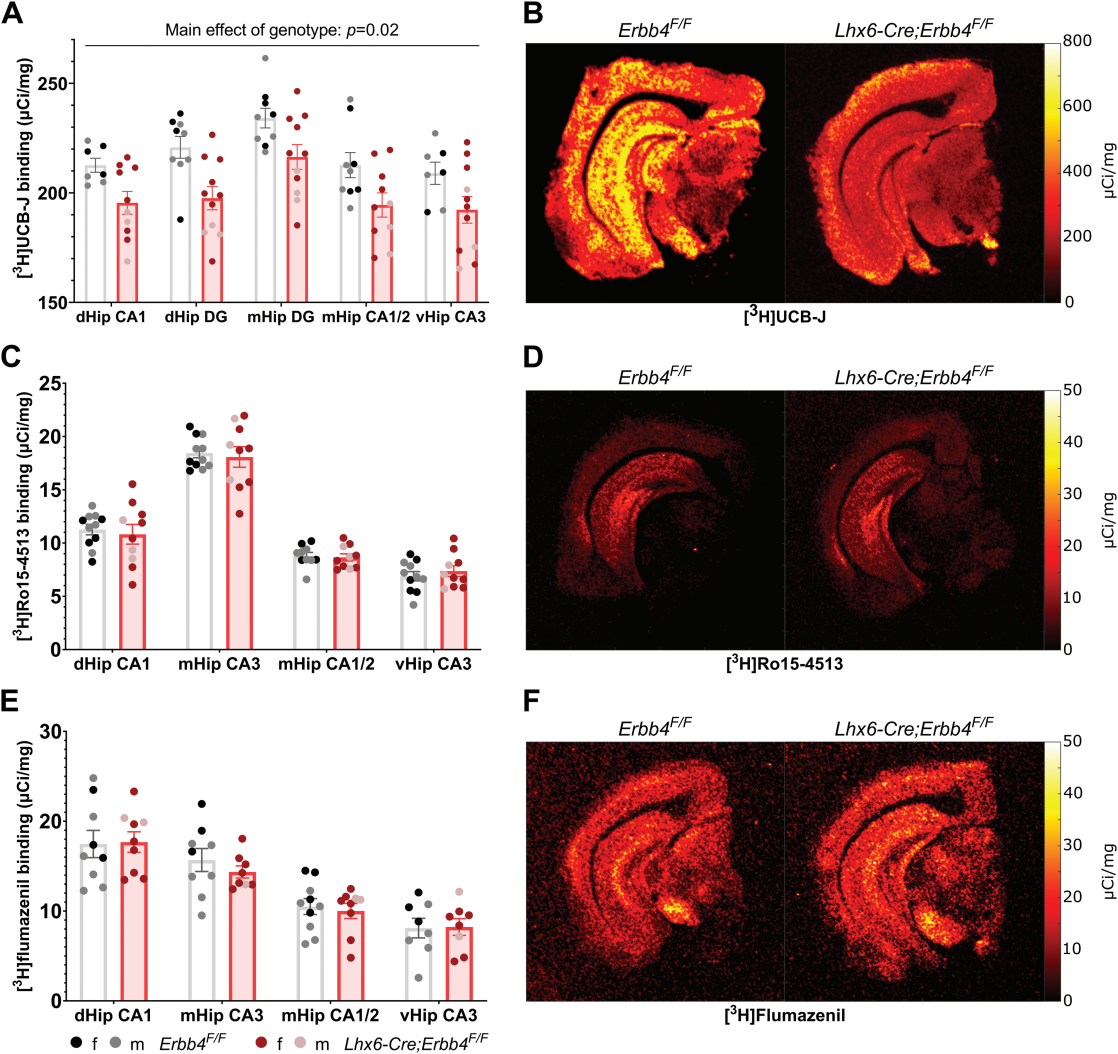
SV2A density, but not α5GABA_A_R or α1-3;5GABA_A_R, is decreased in *Lhx6-Cre;Erbb4*^*F/F*^ mice across the hippocampus. (A) [^3^H]UCB-J autoradiography showed a significant decrease in binding in *Lhx6-Cre;Erbb4*^*F/F*^ mice (*n* = 11, 8 female, 3 male) compared to control animals (*n* = 9, 3 female, 6 male) across all hippocampal ROIs (*P *= .02, η^2^ = 0.29). (B) [^3^H]Ro15-4513 (*Erbb4*^*F/F*^*n* = 11, 5 female, 6 male; *Lhx6;Erbb4*^*F/F*^*n* = 10, 7 female, 3 male) or (C) [^3^H]flumazenil (*Erbb4*^*F/F*^*n* = 10, 4 female, 6 male; *Lhx6;Erbb4*^*F/F*^*n* = 9, 7 female, 2 male) binding did not significantly differ by genotype. dHip CA1: dorsal hippocampus CA1; mHip CA3: middle hippocampus CA3; mHip CA1/2: middle hippocampus CA1/CA2; vHip CA3: ventral hippocampus CA3; dHip DG: dorsal hippocampus dentate gyrus; mHip DG: dentate gyrus.

Autoradiography analysis identified a significant main effect of genotype on [^3^H]UCB-J binding (*F*_(1,18)_ = 7.27, *P *= .02, η^2^ = 0.29), indicating reduced synaptic density in *Lhx6-Cre;Erbb4*^*F/F*^ mice compared to control animals across all hippocampal ROIs (figure 4A). This effect remained significant after adding sex as a covariate of no interest in the analysis (*F*_(1,14)_ = 9.23, *P *= .01, η^2^ = 0.40). No genotype × ROI interaction effect was observed (*F*_(4,66)_ = 0.68, *P* = .61, η^2^ = 0.04).

[^3^H]Ro15-4513 binding, as a measure of α5GABA_A_R density, did not differ significantly between the 2 genotypes (*F*_(1,19)_ = 0.05, *P *= .82, η^2^ < 0.01; figure 4B). Similarly, there were no group differences in α1-3;5GABA_A_R density as measured by [^3^H]flumazenil (*F*_(1,17)_ = 0.07, *P *= .79, η^2^ < 0.01; figure 4C).

## Discussion

In this study, we used conditional *Erbb4* mutants to examine the effects of inhibitory interneuron dysfunction on key neuroimaging markers associated with psychosis in humans. Compared to wild-type mice, *Erbb4* mutants showed increased CBF and glutamine levels in the ventral hippocampus, as well as decreases in SV2A levels across the hippocampus. GABA and glutamate did not significantly differ between the groups, and there were no differences in GABAergic receptor density. Interestingly, in our exploratory analysis of regions outside of the hippocampus (see supplementary tables S2, S4–6, and supplementary Discussion) we found additional group differences, although these did not survive multiple comparisons correction. Overall, our main findings identified abnormalities in hippocampal activity, neurochemistry, and synaptic density that are largely convergent with clinical neuroimaging findings in patients.

Our investigation focused primarily on the hippocampus, based on preclinical evidence suggesting that inhibitory interneuron loss in the ventral part of this region contributes to its hyperactivity and is associated with further electrophysiological and cognitive deficits relevant to psychosis.^2,7^ Indeed, hippocampal disinhibition is suggested to disrupt cognitive functioning in schizophrenia,^7^ consistent with the vital role of inhibitory interneurons in entraining gamma oscillations.^76^ In concordance with the hippocampal hyperactivity hypothesis,^2^ in *Erbb4* mutants it appears that disrupted PV+ interneuron inhibitory control of pyramidal neurons^44^ causes increased neural activity, leading to increased ventral hippocampus CBF, via neurovascular coupling.^18^ Interestingly, our findings align with previous evidence of increased CBV in a schizophrenia-related cyclin D2 knockout mouse model that exhibits inhibitory disruption through PV+ interneuron loss. This corroborates the notion that psychosis-related PV+ interneuron dysfunction leads to aberrantly hyperactive hippocampus in both animal models and in humans. Importantly, our findings that are localized to the ventral part of the hippocampus, match those of increased CBV in psychosis patients^13,15–17^ and CBF in CHR patients^16,25^ in the human anatomical equivalent, the anterior hippocampus. In this context, ventral hippocampal hyperactivity may reflect the differential expression of *Erbb4* and PV+ interneurons from the dorsal (lower) to the ventral (higher) sub-regions of the hippocampus in mice.^43^ In a rodent developmental disruption model of relevance to psychosis, the methylazoxymethanol acetate (MAM) model, projections from a hyperactive ventral hippocampus resulting from inhibitory interneuron disruption drive subcortical hyperdopaminergia,^7^ as well as aberrant network oscillations that are linked to cognitive deficits^77^ via the prefrontal cortex.^7^ Previous characterization of *Erbb4* conditional mutant mice similarly found increased gamma oscillations and disrupted hippocampus-prefrontal theta synchronicity as a result of wide-spread loss of *Erbb4* from primarily PV+ inhibitory interneurons.^44^ Further study of these mice with multimodal imaging combining CBF with electroencephalography and behavior would help to fully characterize these circuit abnormalities.

In terms of ^1^H-MRS findings, we identified an increase in glutamine, with no change in glutamate or GABA, in the ventral hippocampal region. Disinhibition of pyramidal neuronal activity in the *Erbb4* model^42,44,78^ is thought to lead to increased glutamate release.^79,80^ However, glutamine, a precursor of glutamate, has been proposed as a better indicator of glutamatergic neurotransmission.^81^ This is based on the premise that any synaptically released glutamate is quickly taken up by astrocytes and recycled to glutamine.^82,83^ Accordingly, increased glutamine in the medial temporal lobe/hippocampi has previously been detected by ^1^H-MRS in psychosis patients.^33^ Other human studies also showed evidence of elevated Glx^33,34^—a composite measure of glutamate and glutamine that is preferentially measured at the lower magnetic fields such as 1.5 or 3T used in humans, where the separation between those 2 metabolites is not robust.^84^ Our findings thus suggest that increased glutamine may be a good indicator of elevated glutamatergic neurotransmission resulting from inhibitory interneuron dysfunction.

We did not observe an effect of *Erbb4* genotype on hippocampal ^1^H-MRS GABA levels. Previous study of conditional *Erbb4* mutants has identified reduced expression of two GABA synthesizing GAD isomers, GAD65 and GAD67, as well as a reduced frequency of miniature inhibitory postsynaptic GABAergic currents.^44^ However, as a result of a primarily PV+ interneuron disruption, both PV+ interneurons and excitatory pyramidal cells eventually become hyperactive in *Erbb4* mutants, possibly through a compensatory mechanism in order to maintain excitation/inhibition balance.^44^ Such compensatory inhibitory activity may counteract any deficits in GABA synthesis, thereby explaining the lack of measurable differences in GABA between the groups. Indeed, no changes in hippocampal GABA levels were identified in psychosis patients by a previous ^1^H-MRS GABA study.^35^

Contrary to our hypothesis, we found no differences between *Erbb4* mutants and control mice in either α1-3;5GABA_A_R or α5GABA_A_R density. In humans, increases in α1-3;5GABA_A_R availability,^26^ and the more specific decreases in the α5GABA_A_R^27^ subtype, have been identified in groups of antipsychotic-naïve and antipsychotic-free patients with schizophrenia, respectively. Given that *Erbb4* deletion in *Lhx6-Cre* mice primarily affects PV+ interneurons, a lack of α5GABA_A_R changes may be due to this subunit’s putative co-localization with somatostatin-expressing rather than PV+ interneurons,^69^ suggesting that perhaps PV+ interneurons are not associated with the α5GABA_A_R changes seen in humans.^27^ Changes in α1-3;5GABA_A_R in humans are proposed to be the result of compensatory upregulation due to decreased GABAergic release,^5,26^ however how such compensatory increases develop over time is not clear. Future studies should investigate α1-3;5GABA_A_R density and GABA release in *Erbb4* mutants longitudinally, to inform developmental trajectories of inhibitory interneuron dysfunction on GABAergic receptors.

Finally, *post-mortem* findings of decreased dendritic spines and synaptic markers^85–90^ and genetic evidence of variants in synaptic protein coding genes^91–94^ suggest that synaptic dysfunction plays an important role in psychosis pathophysiology. Recent neuroimaging studies have provided *in vivo* evidence for synaptic density decreases in psychosis patients, using [^11^C]UBC-J to image synaptic glycoprotein SV2A,^31,32^ a putative marker of synaptic density.^29,30^ It is known from previous research that synaptic deficits are present in *Erbb4* mutants: excitatory synapses on the fast-spiking inhibitory neurons and presynaptic boutons in chandelier cells,^44^ which are highly expressed in the hippocampus,^95^ are reduced. Our study shows that such synaptic losses can be detected at a macroscopic scale via autoradiography in rodents, and suggest that inhibitory interneuron dysfunction may be underlying the reductions of SV2A observed in patients with psychosis.

While we observed SV2A reductions across all hippocampal sub-regions sampled, significant differences in CBF were only apparent in the ventral hippocampus. This may be a consequence of differences at the synapse or circuit level. Specifically, AMPA and NMDA receptor expression are higher in the CA1 subregion,^96^ potentially contributing to increases in ventral hippocampus perfusion.^16^ Alternatively, the strong bidirectional connections between the ventral hippocampus and the prefrontal cortex^97^—a region high in *Erbb4* expression of PV+ interneurons^44^—may contribute to CBF changes observed here. Future studies may investigate NMDA and AMPA receptor levels in these mice as well as the electrophysiological inputs from the prefrontal region to the ventral hippocampus.

There are some limitations to our study. First, we did not specifically use a *PV-Cre* line to conditionally delete *Erbb4* from specific interneuron sub-types. Instead, an *Lhx6-Cre* line was used to recombine floxed *Erbb4*, targeting interneurons generated in the medial ganglionic eminence.^48^ Of those cell populations, *Erbb4* is more selectively expressed on PV+ interneurons as previously characterized,^42–44^ thus *Lhx6-Cre;Erbb4*^*F/F*^ mice are considered to have a largely PV-specific deletion. Furthermore, due to PV expression appearing relatively later in development compared to *Lhx6* (postnatal vs embryonic, respectively),^98^*PV-Cre* driven *Erbb4*^*F/F*^ recombination does not yield the same alterations in cortical neuron excitability and aberrant gamma oscillations as recombination with *Lhx6-Cre*.^99^ Second, despite known sex differences in psychosis such as incidence rate, age of illness onset, illness course, and treatment response,^100,101^ both male and female mice were used for our study. This was based on following best practice^102,103^ and the 3Rs^104^ to avoid sex bias in preclinical research.^105^ Further, we included sex as a controlling covariate in all our significant analyses. However, further studies with a larger sample and counterbalanced sex ratios would expand on our findings. Third, we did not perform behavioral testing because behavior in *Erbb4* mutants has already been robustly characterized^44–46,106^ and the scope of our study was limited to neuroimaging phenotypes arising from inhibitory interneuron dysfunction. Future studies may expand on these results and link neuroimaging with behavioral readouts to better understand their relationships in the context of this model system. Another limitation is the use of anesthetics to image mice *in vivo*. Isoflurane is known to have effects on the GABA and glutamate system.^107^ However, we used a very low-dose of isoflurane, needed mainly to compensate for the vasoconstrictive effects of medetomidine, the combination which exhibits negligible influence over the GABAergic system.^50,108^

In summary, our study provides direct evidence linking inhibitory interneuron dysfunction in the *Erbb4* mutant mice to analogues of *in vivo* neuroimaging alterations previously identified in psychosis and individuals at clinical high-risk for psychosis. These alterations include increased CBF and glutamine levels, as well as reduced synaptic density, in the hippocampus. Overall, these findings suggest that the use of cross-species neuroimaging methods may be a viable strategy to identify new therapeutic targets and serve as non-invasive measures of target engagement. Furthermore, our findings support the view that targeting inhibitory dysfunction in the hippocampus may be a promising therapeutic strategy for psychosis.

## Supplementary Material

Supplementary material is available at https://academic.oup.com/schizophreniabulletin/.

sbac192_suppl_Supplementary_Material
