# Supplementary material for: Erbb4 Deletion From Inhibitory Interneurons Causes Psychosis-Relevant Neuroimaging Phenotypes
Source: Schizophr Bull. 2022 Dec 27;49(3):569–80. doi: 10.1093/schbul/sbac192 (PMC10154722; doi:10.1093/schbul/sbac192)
Supplement: sbac192_suppl_Supplementary_Material [file sbac192_suppl_supplementary_material.docx]

## SUPPLEMENTARY MATERIALS

**TITLE: Erbb4 deletion from inhibitory interneurons causes psychosis-relevant neuroimaging phenotypes**

Kiemes, A.^1^, Serrano Navacerrada, M. E.^2^, Kim, E.^2^, Randall, K.^2^, Simmons, C.^2^, Rojo Gonzalez, L.^2^, Petrinovic M.M.^3,4^, Lythgoe, D.J.^2^, Rotaru, D.^2^, Di Censo, D.^2^, Hirschler, L.^5,6^, Barbier, E. L.^6^, Vernon, A. C.^3,7^, Stone, J. M.^8^, Davies, C.^1^, Cash, D.^2^*, & Modinos, G.^1,2,3^*

1. Department of Psychosis Studies, Institute of Psychiatry, Psychology and Neuroscience, King’s College London, London, UK
2. Department of Neuroimaging, School of Neuroscience, Institute of Psychiatry, Psychology and Neuroscience, King’s College London, London, UK
3. MRC Centre for Neurodevelopmental Disorders, King's College London, London, UK
4. Department of Forensic and Neurodevelopmental Science, Institute of Psychiatry, Psychology and Neuroscience, King’s College London, London, UK
5. C.J. Gorter Center for High Field MRI, Department of Radiology, Leiden University Medical Center, Leiden, Netherlands; Department of Radiology, Leiden University Medical Center, Leiden, Netherlands
6. Grenoble Institut des Neurosciences, Inserm, Univ. Grenoble Alpes, Grenoble, France
7. Department of Basic and Clinical Neuroscience, School of Neuroscience, Institute of Psychiatry, Psychology and Neuroscience, King’s College London, UK
8. Brighton and Sussex Medical School, University of Sussex, Brighton, UK

* Contributed equally as senior authors

### Supplementary Methods

Non-specific binding for the radioligands [^3^H]Ro15-4513 and [^3^H]flumazenil was negligible (Figure S1). As non-specific binding for [^3^H]UCB-J was minimally present (Figure S1), whole sections were sampled to test for group differences in non-specific binding via independent-samples *t*-test; no group difference was found and subsequently non-specific binding was not subtracted from [^3^H]UCB-J optical densities.


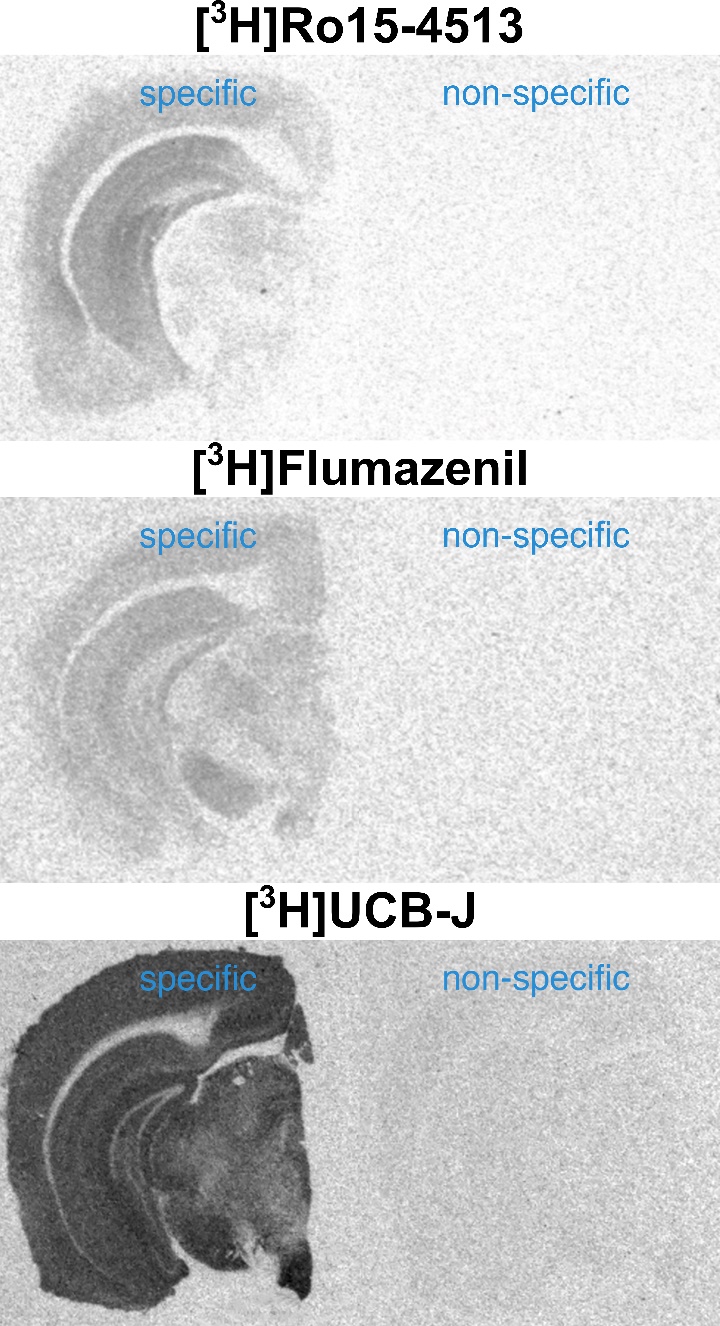


Figure S1. Representative autoradiographs of specific and non-specific binding for [^3^H]Ro15-4513, [^3^H]Flumazenil, [^3^H]UCB-J. Non-specific binding was absent for [^3^H]Ro15-4513 and [^3^H]flumazenil. Non-specific binding was negligible for [^3^H]UCB-J and showed no group difference.

For completeness, in addition to the hippocampus, exploratory analyses were also performed on the amygdala, retrosplenial cortex (RSPcx), visual cortex (Sencx), prelimbic cortex (PLcx), motor cortex (Mcx), and orbital cortex (ORBcx) (Figure S1). Means and standard deviations along with the results of exploratory independent-samples *t*-tests run in GraphPad Prism software (v9.2.0 for Windows) are presented in tables S3-5 below. Due to issues with the tissue preparation, data are missing for ROIs in some mice. Where the number of datapoints per genotype group per ROI fell below n=5, data was not reported.


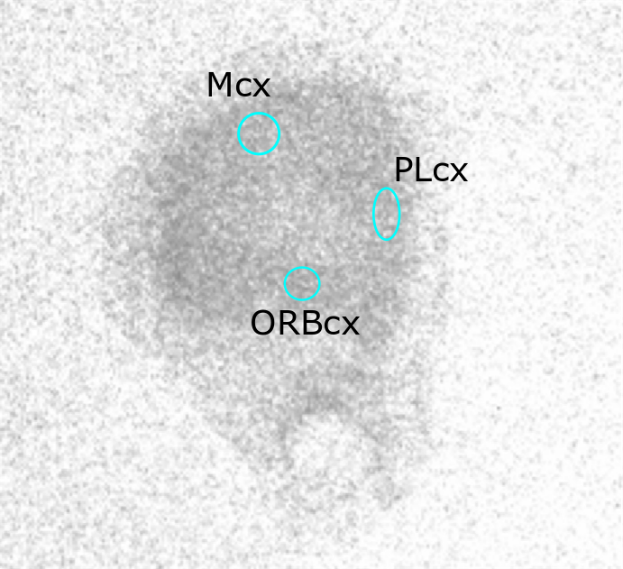

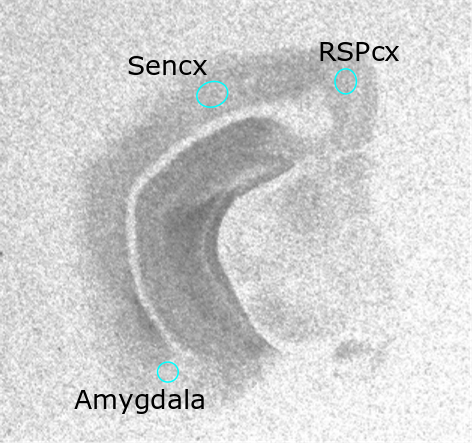


*Figure S2. Representative autoradiograph with additional regions of interest. Frontal regions (left): motor cortex (Mcx), prelimbic cortex (PLcx), orbital cortex (ORBcx). Posterior regions (right): retrosplenial cortex (RSPcx), visual cortex (Sencx), amygdala.*

### Supplementary Results

Table S1. Number of animals and number of females to males per group per measure

|  | | ***Erbb4^F/F^(control)*** | ***Lhx6;Erbb4^F/F^*** |
| --- | --- | --- | --- |
| **Cerebral blood flow** | | 10 (4 f, 6 m) | 12 (9 f, 3 m) |
| **Metabolites** | | 12 (5 f, 7 m) | 12 (9 f, 3 m) |
| **[^3^H]UCB-J** | | | |
|  | dHip CA1 | 7 (2 f, 5 m) | 10 (7 f, 3 m) |
|  | dHip DG | 9 (3 f, 6 m) | 11 (8 f, 3 m) |
|  | mHip DG | 9 (3 f, 6 m) | 11 (8 f, 3 m) |
|  | mHip CA1/2 | 9 (3 f, 6 m) | 10 (7 f, 3 m) |
|  | vHip CA3 | 7 (2 f, 5 m) | 11 (8 f, 3 m) |
| **[^3^H]Ro15-4513** | | | |
|  | dHip CA1 | 11 (5 f, 6 m) | 10 (7 f, 3 m) |
|  | mHip CA3 | 11 (5 f, 6 m) | 10 (7 f, 3 m) |
|  | mHip CA1/2 | 10 (4 f, 6 m) | 10 (7 f, 3 m) |
|  | vHip CA3 | 11 (5 f, 6 m) | 10 (7 f, 3 m) |
| **[^3^H]Flumazenil** | | | |
|  | dHip CA1 | 9 (3 f, 6 m) | 9 (7 f, 2 m) |
|  | mHip CA3 | 9 (3 f, 6 m) | 8 (7 f, 1 m) |
|  | mHip CA1/2 | 10 (4 f, 6 m) | 9 (7 f, 2 m) |
|  | vHip CA3 | 8 (3 f, 5 m) | 8 (6 f, 2 m) |

*Table S2. Cerebral blood flow of whole brain and additional atlas ROIs*

|  | *Erbb4^F/F^****(control)*** | *Lhx6-Cre;Erbb4^F/F^* | *Erbb4^F/F^* vs. *Lhx6-Cre;Erbb4^F/F^* | |
| --- | --- | --- | --- | --- |
|  | Mean (SD) | Mean (SD) | *t* | *p* |
| Amygdala | 0.90 (0.07) | 0.94 (0.09) | 0.98 | 0.34 |
| Brain stem | 0.95 (0.16) | 0.87 (0.28) | 0.82 | 0.42 |
| Cerebellum | 1.13 (0.22) | 1.02 (0.37) | 1.22 | 0.24 |
| Cingulate cx | 1.10 (0.25) | 1.14 (0.19) | 0.40 | 0.69 |
| Colliculus | 1.18 (0.12) | 1.29 (0.22) | 1.32 | 0.20 |
| Entorhinal cx | 1.15 (0.13) | 1.29 (0.14) | 2.40 | 0.03* |
| Hypothalamus | 0.66 (0.06) | 0.67 (0.05) | 0.26 | 0.80 |
| Midbrain | 0.87 (0.07) | 0.83 (0.11) | 1.01 | 0.32 |
| Motor cx | 0.96 (0.14) | 1.00 (0.16) | 0.60 | 0.56 |
| Olfactory | 1.13 (0.09) | 1.19 (0.13) | 1.23 | 0.23 |
| PAG | 0.76 (0.11) | 0.74 (0.13) | 0.28 | 0.78 |
| Pallidum | 0.92 (0.05) | 0.95 (0.08) | 0.92 | 0.37 |
| Prefrontal cx | 1.05 (0.16) | 1.11 (0.12) | 0.98 | 0.34 |
| Sensory cx | 1.08 (0.13) | 1.16 (0.14) | 1.39 | 0.18 |
| Septum | 0.60 (0.15) | 0.62 (0.12) | 0.46 | 0.65 |
| Striatum | 0.92 (0.09) | 0.95 (0.09) | 0.57 | 0.57 |
| Thalamus | 0.87 (0.08) | 0.92 (0.14) | 1.13 | 0.27 |
| ROI: region of interest; cx: cortex; PAG: periaqueductal gray; **p*<0.05 | | | | |


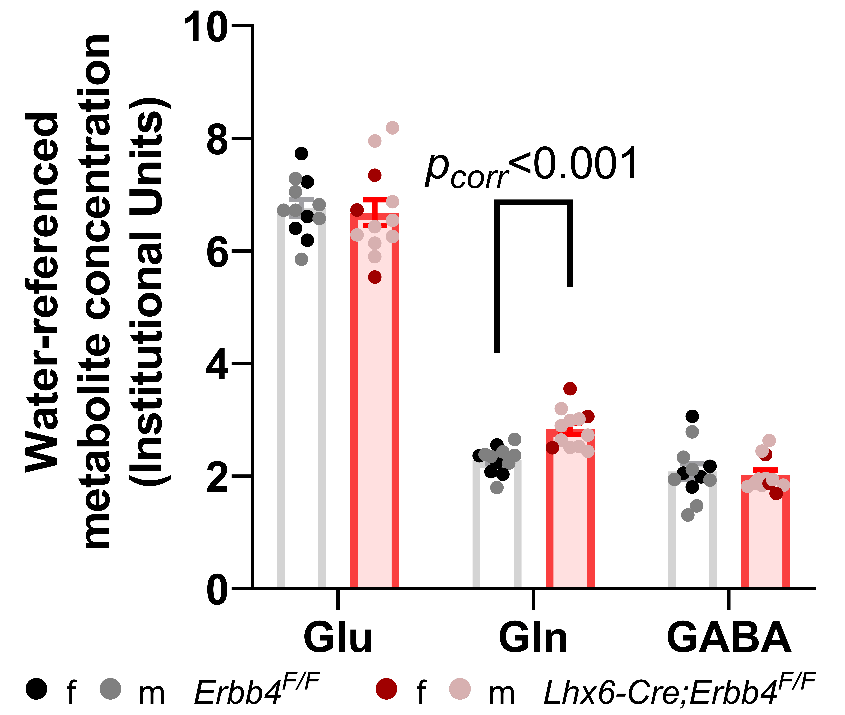


Figure S3. Water-referenced ^1^H-MRS metabolite concentrations in the ventral hippocampus. Glutamine was significantly higher in Lhx6-Cre;Erbb4^F/F^ mutant mice (n=12, 9 female, 3 male) compared to control mice (n=12, 5 female, 7 male; p_corr_<0.001, d=1.96). GABA: gamma-aminobutyric acid; Glu: glutamate; Gln: glutamine.

*Table S3. ^1^H-MRS quality control parameters*

|  | *Erbb4^F/F^****(control)*** | *Lhx6-Cre;Erbb4^F/F^* | *Erbb4^F/F^* vs. *Lhx6-Cre;Erbb4^F/F^* | |
| --- | --- | --- | --- | --- |
|  | Mean (SD) | Mean (SD) | *t* | *p* |
| SNR | 14.7 (1.7) | 15.8 (3.8) | 0.87 | 0.39 |
| FWHM (ppm) | 0.05 (0.01) | 0.05 (0.03) | 0.60 | 0.55 |
| GABA CRLB (%) | 10.58 (2.39) | 10.92 (2.94) | 0.31 | 0.76 |
| Gln CRLB (%) | 9.50 (1.73) | 8.75 (4.07) | 0.59 | 0.01* |
| Glu CRLB (%) | 4.08 (0.67) | 4.00 (1.13) | 0.22 | 0.10 |
| SNR: signal-to-noise ratio; FWHM: full width half maximum; CRLB: Cramér-Rao lower bound; Gln: glutamine; **p*<0.05 | | | | |

*Table S4. Exploratory independent t-tests of additional [^3^H]Ro15-4513 ROIs*

|  | *Erbb4^F/F^****(control)*** | *Lhx6-Cre;Erbb4^F/F^* | *Erbb4^F/F^* vs. *Lhx6-Cre;Erbb4^F/F^* | |
| --- | --- | --- | --- | --- |
|  | Mean (SD)  (µCi/mg) | Mean (SD)  (µCi/mg) | *t* | *p* |
| Amygdala | 3.07 (0.95) | 3.11 (1.15) | 0.09 | 0.93 |
| RSPcx | 2.91 (1.08) | 3.91 (0.86) | 2.33 | 0.03* |
| Sencx | 5.7 (1.19) | 5.94 (1.56) | 0.39 | 0.69 |
| PLcx | 7.27 (1.89) | 6.1 (1.97) | 1.35 | 0.19 |
| Mcx | 5.54 (1.28) | 6.37 (1.18) | 1.45 | 0.16 |
| ORBcx | 3.88 (2.1) | 4.98 (1.37) | 1.35 | 0.19 |
| ROI: region of interest; RSPcx: retrosplenial cortex; Sencx: visual cortex; PLcx: prelimbic cortex; Mcx: motor cortex; ORBcx: orbitofrontal cortex; **p*<0.05 | | | | |

*Table S5. Exploratory independent t-tests of additional [^3^H]flumazenil ROIs*

|  | *Erbb4^F/F^****(control)*** | *Lhx6-Cre;Erbb4^F/F^* | *Erbb4^F/F^* vs. *Lhx6-Cre;Erbb4^F/F^* | |
| --- | --- | --- | --- | --- |
|  | Mean (SD)  (µCi/mg) | Mean (SD)  (µCi/mg) | *t* | *p* |
| Amygdala | 5.19 (2.62) | 7.23 (4.6) | 1.02 | 0.32 |
| RSPcx | 10.75 (4.05) | 7.87 (1.68) | 1.99 | 0.06 |
| Sencx | 15.97 (4.2) | 12.75 (2.05) | 1.97 | 0.07 |
| PLcx | 13.01 (3.75) | 12.36 (3.55) | 0.36 | 0.72 |
| Mcx | 14.19 (3.4) | 13.11 (2.82) | 0.73 | 0.47 |
| ORBcx | 12.96 (5.07) | 11.9 (3.77) | 0.46 | 0.65 |
| ROI: region of interest; RSPcx: retrosplenial cortex; Sencx: visual cortex; PLcx: prelimbic cortex; Mcx: motor cortex; ORBcx: orbitofrontal cortex | | | | |

*Table S6. Exploratory independent t-tests of all [^3^H]UCB-J ROIs*

|  | *Erbb4^F/F^****(control)*** | *Lhx6-Cre;Erbb4^F/F^* | *Erbb4^F/F^* vs. *Lhx6-Cre;Erbb4^F/F^* | |
| --- | --- | --- | --- | --- |
|  | Mean (SD)  (µCi/mg) | Mean (SD)  (µCi/mg) | *t* | *p* |
| RSPcx | 192.09 (14.95) | 176.92 (20.94) | 1.80 | 0.09 |
| Sencx | 197.47 (14.00) | 189.69 (15.12) | 1.18 | 0.25 |
| PLcx | 195.91 (11.97) | 193.83 (20.44) | 0.24 | 0.82 |
| Mcx | 191.07 (15.86) | 181.08 (12.27) | 1.54 | 0.14 |
| ORBcx | 192.52 (13.61) | 179.93 (21.48) | 1.31 | 0.21 |
| ROI: region of interest; RSPcx: retrosplenial cortex; Sencx: visual cortex; PLcx: prelimbic cortex; Mcx: motor cortex; ORBcx: orbitofrontal cortex | | | | |

### Supplementary Discussion

Exploratory analysis of CBF values in additional ROIs revealed a significant increase in CBF in the entorhinal cortex of *Erbb4* mutants. Although no brain perfusion studies have implicated the entorhinal cortex in psychosis, multiple lines of evidence converge to implicate this area in relation to inhibitory interneurons and cognitive deficits. The entorhinal cortex receives major outputs from hippocampal regions such as the CA1 and subiculum^1^. Further, preclinical evidence indicates that entorhinal cortex communication with the hippocampus is integral for gamma oscillations^2^, prepulse inhibition is dependent on this region^3^, and a genetic animal model (LPA1-deficient mice) shows reduction of entorhinal cortex PV+ interneurons^4^. Previous work in *Lhx6-Cre;Erbb4^F/F^* mice reported inhibitory synapse deficits in the lateral entorhinal cortex, which were thought to contribute to hypersynchronicity between the entorhinal cortex and the trisynaptic circuit (hippocampal DG to CA3 to CA1)^5^ in this mouse mutant. Our findings demonstrate that inhibitory interneuron disruption also increases network activity in the entorhinal cortex in psychosis in *Erbb4* mutant mice.

Interestingly, our exploratory analysis revealed (uncorrected) α5GABA_A_R increases and a trend toward decreases of α1-3;5GABA_A_R in the retrosplenial cortex. [^3^H]Flumazenil and [^3^H]Ro15-4513 binding distributions seem to be anticorrelated^6^ potentially yielding such differential results in the same region. The retrosplenial cortex features reciprocal links to the hippocampus, parahippocampus and thalamus and is involved in an array of cognitive processes including spatial navigation, memory, and planning^7^. While neuroimaging research has identified functional connectivity changes psychosis patients suggesting aberrant activity^8–11^, GABAergic receptors have not yet been studied in the retrosplenial cortex. Nonetheless, preclinical work suggests that there might be a decrease in PV+ interneurons in the retrosplenial cortex in relation to psychosis^12^, and here we extend these findings to suggest that inhibitory interneuron impairment in *Erbb4* mutants generates subtle GABAergic receptor differences that may contribute to aberrant neural activity.

## Supplementary References

1. Witter MP, Naber PA, van Haeften T, et al. Cortico-hippocampal communication by way of parallel parahippocampal-subicular pathways. *Hippocampus*. 2000;10(4):398-410. doi:10.1002/1098-1063(2000)10:4<398::AID-HIPO6>3.0.CO;2-K

2. Fernández-Ruiz A, Oliva A, Nagy GA, Maurer AP, Berényi A, Buzsáki G. Entorhinal-CA3 Dual-Input Control of Spike Timing in the Hippocampus by Theta-Gamma Coupling. *Neuron*. 2017;93(5):1213-1226.e5. doi:10.1016/j.neuron.2017.02.017

3. Goto K, Ueki A, Iso H, Morita Y. Reduced prepulse inhibition in rats with entorhinal cortex lesions. *Behav Brain Res*. 2002;134(1-2):201-207. doi:10.1016/s0166-4328(02)00039-6

4. Cunningham MO, Hunt J, Middleton S, et al. Region-Specific Reduction in Entorhinal Gamma Oscillations and Parvalbumin-Immunoreactive Neurons in Animal Models of Psychiatric Illness. *J Neurosci*. 2006;26(10):2767-2776. doi:10.1523/JNEUROSCI.5054-05.2006

5. Del Pino I, Garcia-Frigola C, Dehorter N, et al. Erbb4 deletion from fast-spiking interneurons causes schizophrenia-like phenotypes. *Neuron*. 2013;79(6):1152-1168. doi:10.1016/j.neuron.2013.07.010

6. Lingford-Hughes A, Hume SP, Feeney A, et al. Imaging the GABA-Benzodiazepine Receptor Subtype Containing the α5-Subunit In Vivo with [11C]Ro15 4513 Positron Emission Tomography. *J Cereb Blood Flow Metab*. 2002;22(7):878-889. doi:10.1097/00004647-200207000-00013

7. Vann SD, Aggleton JP, Maguire EA. What does the retrosplenial cortex do? *Nat Rev Neurosci*. 2009;10(11):792-802. doi:10.1038/nrn2733

8. Tendolkar I, Weis S, Guddat O, et al. Evidence for a dysfunctional retrosplenial cortex in patients with schizophrenia: a functional magnetic resonance imaging study with a semantic—perceptual contrast. *Neuroscience Letters*. 2004;369(1):4-8. doi:10.1016/j.neulet.2004.07.024

9. Siemerkus J, Irle E, Schmidt-Samoa C, Dechent P, Weniger G. Egocentric spatial learning in schizophrenia investigated with functional magnetic resonance imaging. *NeuroImage: Clinical*. 2012;1(1):153-163. doi:10.1016/j.nicl.2012.10.004

10. Wang Y, Yan C, Yin D zhi, et al. Neurobiological changes of schizotypy: evidence from both volume-based morphometric analysis and resting-state functional connectivity. *Schizophr Bull*. 2015;41 Suppl 2:S444-454. doi:10.1093/schbul/sbu178

11. Bluhm RL, Miller J, Lanius RA, et al. Retrosplenial cortex connectivity in schizophrenia. *Psychiatry Research: Neuroimaging*. 2009;174(1):17-23. doi:10.1016/j.pscychresns.2009.03.010

12. Klimczak P, Rizzo A, Castillo-Gómez E, et al. Parvalbumin Interneurons and Perineuronal Nets in the Hippocampus and Retrosplenial Cortex of Adult Male Mice After Early Social Isolation Stress and Perinatal NMDA Receptor Antagonist Treatment. *Frontiers in Synaptic Neuroscience*. 2021;13. https://www.frontiersin.org/article/10.3389/fnsyn.2021.733989. Accessed March 4, 2022.
